# Single-cell sequencing reveals the cell map and transcriptional network of sporadic vestibular schwannoma

**DOI:** 10.3389/fnmol.2022.984529

**Published:** 2022-10-11

**Authors:** Chu Yidian, Lin Chen, Deng Hongxia, Li Yanguo, Shen Zhisen

**Affiliations:** ^1^The Affiliated Lihuili Hospital, Ningbo University, Ningbo, China; ^2^School of Medicine, Ningbo University, Ningbo, China; ^3^Institute of Drug Discovery Technology, Ningbo University, Ningbo, China

**Keywords:** sporadic vestibular schwannoma, single-cell transcriptomics, differentially expressed genes, immune cell infiltration, Schwann cells

## Abstract

In this study, based on three tumor samples obtained from patients with sporadic vestibular schwannoma, 32,011 cells were obtained by single-cell transcriptome sequencing, and 22,309 high-quality cells were obtained after quality control and double cells removal. Then, 18 cell clusters were obtained after cluster analysis, and each cluster was annotated as six types of cells. Afterward, an in-depth analysis was conducted based on the defined six cell clusters, including characterizing the functional characteristics of each cell subtype, describing the cell development and differentiation pathway, exploring the interaction between cells, and analyzing the transcriptional regulatory network within the clusters. Based on these four dimensions, various types of cells in sporadic vestibular schwannoma tumor tissues were described in detail. For the first time, we expanded on the functional state of cell clusters that have been reported and described Schwann cells in the peripheral nervous system, which have not been reported in previous studies. Combined with the data of sporadic vestibular schwannoma and normal tissues in the gene expression omnibus (GEO) database, the candidate biomarkers of sporadic vestibular schwannoma were explored. Overall, this study described the single-cell map of sporadic vestibular schwannoma for the first time, revealing the functional state and development trajectory of different cell types. Combined with the analysis of data in the GEO database and immunohistochemical verification, it was concluded that HLA-DPB1 and VSIG4 may be candidate biomarkers and potential therapeutic targets for patients with sporadic vestibular schwannoma.

## Introduction

Vestibular schwannoma (VS), also known as acoustic neuroma, accounts for 8–10% of intracranial benign tumors and approximately 80–90% of cerebellopontine angle tumors (Zou and Hirvonen, 2017). There are two major classification of VS, based on the side of the internal auditory canal involved: unilateral and bilateral. In most cases, unilateral tumors presenting as sporadic lesions constitute most VSs; bilateral tumors constitute only 4–6% of these lesions and are considered NF2 related schwannomatosis (Plotkin et al., 2022). Among them, the loss of the function of merlin, a tumor suppressor protein encoded by NF2 gene, is an important step in the pathogenesis of schwannoma, and the biallelic mutation of NF2 is also found in some sporadic VSs (Bachir et al., 2021).

At present, surgical treatment is still an indisputable treatment for large VS tumors, whereas small and medium-sized tumors can be treated by repeated MRI observation, surgery, or radiotherapy. In addition, targeted drug therapy is considered as a treatment that can control tumor growth as well as avoid facial nerve function damage and other poor prognosis caused by surgery. However, unlike neurofibromatosis type II-related VS, for which there are monoclonal antibody angiogenesis inhibitors targeting the vascular endothelial growth factor (VEGF), sporadic VS has no suitable drug treatment so far, which is related to the great variability in the growth rate and size of sporadic VS (Taurone et al., 2015). In recent years, in-depth study of sporadic VS has revealed that some proteins, inflammatory cytokines, miRNA, tissue proteins, and cerebrospinal fluid (CSF) components that regulate the conformational changes of merlin are closely related to the increase in tumor volume (Ariyannur et al., 2018; de Vries et al., 2019; Huang et al., 2019b). Previous studies are based on traditional Bulk RNA-Seq or immunohistochemical methods, which obviously ignore the heterogeneity of this tissue structure. Therefore, although the microenvironment may play a key role in the occurrence of sporadic VS, the exact cell types and factors involved are still unclear. One of the main limitations in understanding the biological role of the microenvironment in VS is the lack of markers to distinguish its cell types. However, with the advent of single-cell transcriptome analysis (scRNA-Seq), the cell type composition of tissues can presently be determined without bias, providing a powerful tool for exploring genetic and functional heterogeneity, detecting rare cell clusters, and reconstructing evolutionary lineages. At present, scRNA-Seq has been successfully applied to the study of breast cancer (Ding et al., 2020), head and neck tumors (Qi et al., 2019), colorectal cancer (Tieng et al., 2020), pancreatic cancer and other cancers (Luo et al., 2020).

Herein, we applied scRNA-Seq to the tumor tissues of three VS cases to evaluate the cellular and molecular composition of the tumor microenvironment. A total of 32,011 cells were obtained in three cases, and 22,309 high-quality cells were obtained after quality control and double cells removal. Six cell types corresponding to 18 clusters were identified by single-cell sequencing, including Schwann cells, myeloid cells, fibroblasts, lymphocytes, and endothelial cells. Sporadic VS cell subgroup cluster analysis was conducted for the first time, which provided a complete sporadic VS cell map and revealed the differential gene expression of each cell type, as well as the functional status and development trajectory of myeloid cells, lymphocytes, and stromal cells. In addition, ligand–receptor analysis showed that there was a wide relationship between fibroblasts and immune cells, indicating that stromal cells play an important role in tumor proliferation and growth. After bioinformatics analysis of the data of sporadic VS and normal tissues downloaded from the gene expression omnibus (GEO) database, 131 Differentially Expressed Genes (DEGs) and three hub genes were found and identified. They hub genes may be candidate biomarkers of sporadic VS, which provide a new idea for the further study of the molecular mechanism of sporadic VS and the screening of drug targets.

## Materials and methods

### Patient information and specimen collection

Ethical approval for this study was issued by the Ethics Committee of Li Huili Hospital in Ningbo (Ningbo, China). Samples were collected from patients with unilateral sporadic VS diagnosed by Li Huili in Ningbo, China, after obtaining their informed consent. One tumor specimen per individual was excised from the CPA *via* the translabyrinthine approach. One-half of each tissue specimen was analyzed by using scRNA-Seq; the other half of each tissue specimen was fixed in formalin and embedded in paraffin for subsequent immunohistochemical analysis. For each patient, cell viability was verified using trypan blue, and the library was prepared for approximately 90% of the cells, with high cell activity in each sample.

### Single-cell transcriptome analysis library preparation, sequencing, and alignment

According to the manufacturer’s instructions, the prepared cell suspension was used for a 10x Chromium Single Cell 3′ library with Chromium Single Cell 30 v3 (10 X Genomics, 2019). Each sample contained approximately 8,000 cells. Follow-up sequencing was performed using Illumina (Nova 6000), according to the manufacturer’s instructions.

### Single-cell transcriptome analysis data preprocessing and quality control

Cell Ranger Software Suite (version 3.1.0) was used to process the single-cell transcriptome data by performing alignment, filtering, barcode separation, and UMI counting using default parameters. Cell Ranger was used to align the original readings with the human reference genome *GRCh38* (Jindal et al., 2018). The characteristic bar code matrix of each sample was generated for secondary analysis. For quality control, each sample was initially analyzed using a scDblFinder R package.^1^ Then, the cells were filtered using the following criteria: (1) 500 < nFeature RNA < 5,000; (2) 1,000 ≤ nCount RNA ≤ 20,000; (3) percentage of mitochondrial genes < 30%; (4) percentage of hemoglobin gene < 0.01%. Mitochondrial and ribosomal genes were removed. After filtering, a total of 22,309 high-quality cells were obtained from the three patients’ samples, and an integrated pipeline with a default Seurat R package was used for further integration analysis.

### Unsupervised cell type annotation and non-linear dimensionality reduction of subtypes (t-stochastic neighbor embedding/unified manifold approximation and projection) recognition clustering and cell type annotation

In this paper, Principal components analysis (PCA) was used to reduce the dimension of the data, and the FindClusters function of Seurat package was used for primary cell clustering analysis. The visual clustering results were presented by unified manifold approximation and projection (UMAP) dimensionality reduction analysis. In this study, the cells were divided into 18 subgroups. According to the marker genes in the CellMarker database, the cell clusters in this experiment were annotated by single-cell sequencing cell type annotation software SingleR, and 18 cell clusters were annotated as six types of cells. We selected the marker genes from the published literature to identify the six types of cells, such as GAP43 and MPZ for Schwann cells (Liu et al., 2015), LYZ and CD14 for myeloid cells (Chen et al., 2020), COL1A1 and COL1A2 for CAFs (Chen et al., 2020), CD2, CD3D, CD3E, and CD3G for NK/T cells, VWF and CLDN5 for Endothelial cells (Bassez et al., 2021; Olbrecht et al., 2021), CD79 and CD79A for B cells (Mason et al., 1995).

The FindAllMarkers function with the following parameters was used to calculate the marker genes of all cells in each cell cluster and their corresponding cluster: only positive markers, and fraction of expressed cells in the cluster ≥ 0.25. Functional analysis of the cell cycle stage was scored according to the implementation in “Seuratv3.” The AddModuleScore function was used to score the gene set expression of the Broad Institute Hallmark signature set and the upregulated and downregulated genes of M1 and M2. The R package “progeny” was used to calculate the activity score of the carcinogenic signal pathway.

### Pathway enrichment analysis

Genomic variation analysis of myeloid cells, Schwann cells, NK/T cells, and stromal cells (GSVA; package version 1.34.0) was performed to determine the genomes that were significantly enriched in each cluster. All genomes were downloaded from the database MSigDB^2^ and analyzed by GSVA and GO (clusterProfiler package version 3.14.3) to identify the biological processes (BP) that were significantly enriched in each subtype.

### Pseudotime trajectory construction

In the dimension of cell development and progress, we constructed the development trajectory of the Schwann cell cluster, explored the development and differentiation trajectory of this group of cells and the heterogeneity within the cell, and found the differentiation potential of SC-C5 and SC-C6.

### Communication between cells

Potential paracrine interactions were calculated using the “CellPhoneDB” R package with default parameters, and intercellular communication based on ligands and receptors was analyzed. *P* < 0.05 was used as the cutoff value to filter ligand–receptor pair data. Then, the data with biological significance were selected for display. It was speculated that tumor fibroblasts may play a role in promoting the occurrence and development of tumor tissue in VS.

### Gene expression omnibus database verification

The sporadic VS-related dataset GSE141801 (36 sporadic vestibular schwannomas and seven normal tissue samples) was downloaded from the GEO database. GSE54934 (31 sporadic vestibular schwannomas and three normal meningeal tissue samples) only selected sporadic VS and normal nerve tissue samples for follow-up analysis. The DEGs were identified and intersected with the DEGs identified in this study. A total of 131 DEGs were identified [with absolute log2 multiple variation (FC) > 1 and adjusted *P* < 0.05]. The abundant functional pathways of the DEGs included the complement and coagulation cascade, *Staphylococcus aureus* infection, myelin structure formation, activation of MHC class II receptor activity, and so on. Three hub genes were also identified. BP analysis showed that these genes were mainly enriched in intestinal immune network IgA, lipid metabolism, brain development, and other pathways. BioPortal^3^ online platform was used to analyze the gene network and its co-expressed genes. Toscape’s Biological Networks Gene Oncology tool (BiNGO) (version 3.0.3) plug-in was used to analyze and visualize the BP of the central genes.

### Immunohistochemical analysis

We used the Envision two-step method for immunohistochemical staining of HLA-DPB1 (rabbit, Abcam, CHINA, EPR11226) and VSIG4 (rabbit, Abcam, CHINA, EPR22576-70). To put it simply, the tissue sections were incubated in EDTA antigen repair solution at 100°C for 20 min, then cooled at room temperature to prepare the sample. The immunohistochemical staining results were evaluated by two pathologists using a double-blind method.

### Statistical analysis

All statistical analyses were performed using R package version 4.1.2.^4^ Due to the fact that the overall distribution is masked by a large number of data points, not all violin charts show each data point. *P* < 0.05 was considered to show a statistically significant difference.

## Results

### The whole landscape of sporadic vestibular schwannoma cells

**FIGURE 1:**
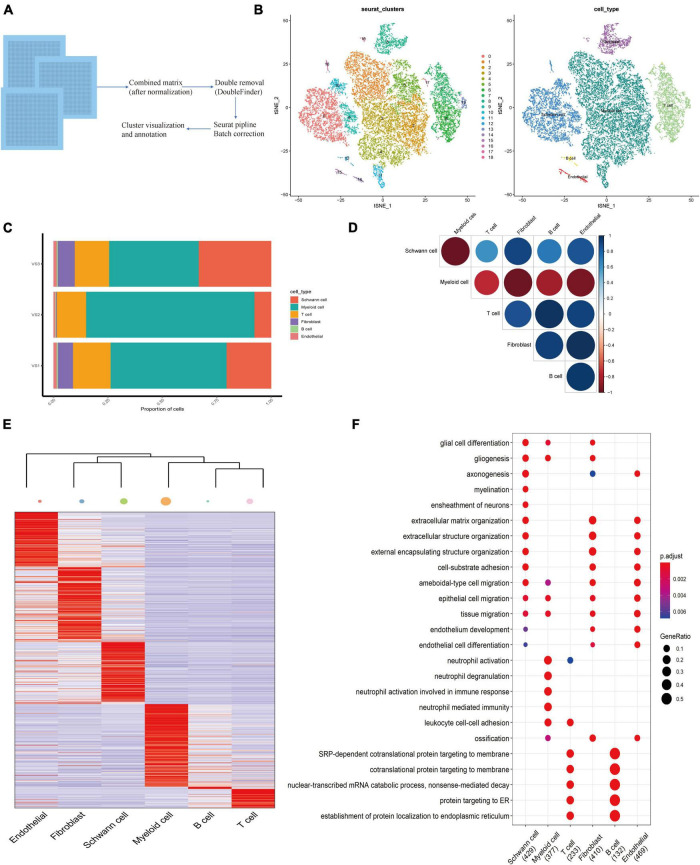
Establishing of the Cell Atlas for sporadic Vestibular Schwannoma Based on Single-Cell RNA-Seq. **(A)** An overview of the study design and workflow. **(B)** Overall cell type composition of 224,309 cells from three sporadic Vestibular Schwannoma patients were visualized with TSNE projection. **(C)** Correlation circle diagram of 6 subgroups. **(D)** The heat map shows differentially expressed genes for six cell types in VS. The color keys from purple to yellow indicate low to high expression levels. Typical cell marker gene expression that defines each cluster is shown at the right. **(E)** The heatmap shows the hierarchical clustering relationship of each cell type, and the size of the point represents the number of cell subsets. **(F)** The bubble diagram shows the functional enrichment diagram of 6 cell types.

Fresh samples of three patients with pathologically diagnosed VS were obtained. All patients were diagnosed as unilateral sporadic VS and did not receive chemotherapy or radiotherapy before tumor resection. After preliminary quality control of the overall characteristics of the sporadic VS (Supplementary Figures 1A,B). We obtained 9,877 cell transcripts from tumor tissue samples, and a total of 22,309 genes were detected. We use Seurat software to preprocess the data and analyze the single-cell data (Figure 1A). Finally, through principal component analysis, we identified 18 major cell clusters (Figure 1B). According to the typical cell markers listed (Supplementary Figure 1D), 12,283 cells in groups 1–5 and 11 were classified as myeloid cells, accounting for 55.10% of the total number of cells. A total of 4,904 cells in group 0, 9, 10, and 16 were identified as Schwann cells. In addition, there are lymphocytes (16.2%), fibroblasts (5.53%), and endothelial cells (279) (1.30%). The clustering results after visual dimensionality reduction by UMAP (Supplementary Figure 1B) showed that the cells from different sample sources of VS did not cluster separately, indicating that the data integration method was effective and removed the batch effect between samples. The cell composition of the three groups of samples in VS1, VS2, and VS3 is similar (Figure 1C). The expression levels of marker genes in six cell clusters were depicted by thermography, and the marker genes are shown in (Supplementary Table 1 and Figure 1D). GSVA analysis of differential genes showed that these genes were mainly enriched in asthma, autoimmune thyroid disease, rheumatoid arthritis, and other disease pathways (Supplementary Figure 1E). Based on the correlation analysis of each subgroup, a strong correlation of myeloid cells with Schwann cells, fibroblasts, and endothelial cells was found (Figures 1F,G).

### Heterogeneity of Schwann cell clusters in vestibular schwannoma

**FIGURE 2:**
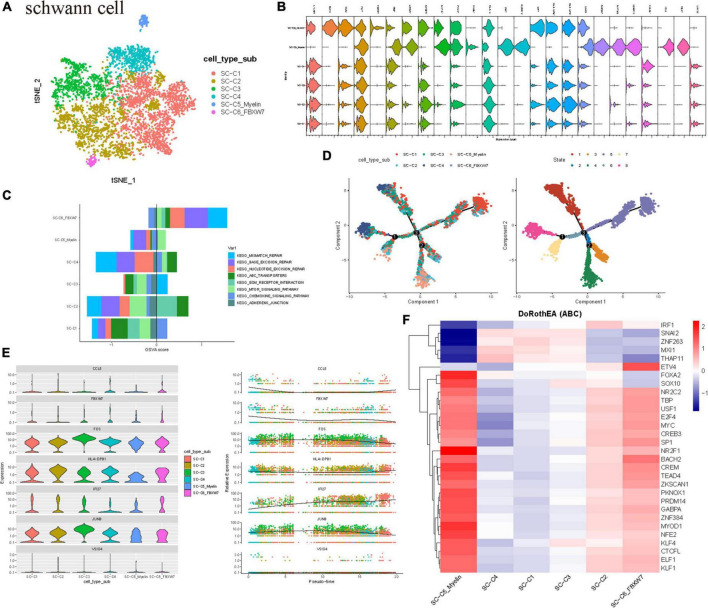
Schwann cell population heterogeneity in sporadic acoustic neuroma. **(A)** TSNE diagram showing Schwann cell subsets. Different cell types are indicated by different colors. **(B)** Violin plots showed the scaled expression levels of canonically cell marker genes used to identify each cell types. **(C)** The histogram shows GSVA score of the first 8 KEGG enrichment pathways. **(D)** All SCs ordered along pseudotime trajectories, with the cells color-coded by cluster. **(E,C)** Subtypes. The left dendrogram was calculated using all differential genes and is colored according to group membership. **(E)** Scatter plots depicting scores for the indicated upstream regulators for each cell across pseudotime. X axis, cell subset; Y axis, Relative Expression.**(F)** Heatmap of top 50 highly variable TF activities among the Schwann cell subsets; the z-scores of TF activities are coded.

Current studies have shown that Schwann cells participate in the process of fragment removal, axon and myelin regeneration, and target organ reinnervation after peripheral nerve injury. Schwann cells are rapidly activated into the repair process after peripheral nerve injury. Schwann cells undergo a series of dynamic cell remodeling changes, transform into repair phenotypes, promote nerve regeneration, guide reinnervation of target organs, and thus restore nerve function, in which there are many signal pathways. These processes are regulated by transcriptional regulators (Abdo et al., 2019). Furthermore, to identify the cell clusters and molecular markers related to the tumor proliferation of sporadic VS, we subdivided the Schwann cell population into six subgroups of C1–C6 (Figures 2A,B). Based on the gene markers and the high expression of HLA-DPB1, S100A6, HLA-DRA, and other genes related to antigen presentation in GSVA, SC-C1 and SC-C3 cells were mainly enriched in hematopoiesis, suggesting that SC-C3 cells may be related to tumor vascular proliferation (Supplementary Figure 2C). It is worth noting that SC-C3 is also significantly enriched in the MAPK signaling pathway. Previous studies have shown that this pathway is activated after peripheral nerve injury and is the key signal pathway to initiate dedifferentiation/transdifferentiation of Schwann cells. Continuous activation of the MAPK/ERK signaling pathway in normal peripheral nerves can lead to demyelination and induce an inflammatory response (Napoli et al., 2012). After peripheral nerve injury, p38MAPK can regulate the elongation and arrangement of Schwann cells around axons to meet the needs of myelination (Newbern et al., 2011). Upregulating the expression of transcription factor c-Jun can promote the demyelination of Schwann cells and make Schwann cells transdifferentiate into a repair-like morphology. SC-C4 showed chemokine activity. SC-C5_myelin has the function of myelination, promoting the survival of damaged neurons and axon regeneration. SC-C5 expresses cell matrix markers (including mitochondrial gene MT-ATP6, ILI27, which is closely related to lupus erythematosus), and is rich in the process of extracellular matrix and axon regeneration. Most cells of SC-C5_myelin is in G2 phase (Supplementary Figure 2A). Therefore, we speculate that SC-C5 and SC-C6 are necessary cell groups to maintain the normal biological function of vestibular nerve myelin sheath tissue. SC-C5 specifically expresses NCMAP (non-dense myelin associated protein), PRX, and other proteins involved in the maintenance of the peripheral nerve myelin sheath. SC-C6 specifically expresses FBXW7. As one of the important recognition factors of the ubiquitin proteasome degradation pathway, most of the substrates of FBXW7 are oncogenes, such as cyclin E, c-MYC, c-JUN, and Notch, which are degraded through the FBXW7-mediated ubiquitin proteasome pathway. FBXW7 mutations and deletions can cause the accumulation of these genes related to cancer cell proliferation and have been found in ovarian cancer, breast cancer, and colorectal cancer (Harty et al., 2019). In this study, biological experiments were not used to clarify the specific function and mechanism of these key genes, which still need to be verified by follow-up experiments.

Therefore, it is obvious that there is heterogeneity in the expression profile and function of Schwann cells, and each subgroup has different differentiation, proliferation, and immunocyte chemotaxis. The GSVA scores of DNA damage repair pathways such as mismatch repair, base excision repair, nucleotide cleavage repair, and ABC transporter were higher in SC-C6 Schwann cells (Figure 2C).

The composition of Schwann cell clusters in the three patients was also heterogeneous, and the cell cycle revealed that C5 clusters were in the active phase of proliferation. To further study the origin, differentiation, and development of Schwann cells using the data, the trajectory of Schwann cells was analyzed. The results showed that there were mainly SCC5_myelin and SC-C6 cells (Figure 2D) at the beginning of the developmental trajectory. We observed three distinct evolutionary processes of Schwann cells, which indicate that some SC-C5_myelin and SC-C6 cells have the ability to differentiate into other cell subtypes (Figure 2E).

Then, the TF of Schwann cells was determined by DoRothEA package analysis. It was also found that the C5 clusters expressed several tumor-related highly active transcription factors, such as FOXA2, which has been proven to be highly related to tumorigenesis and malignant transformation and is involved in the regulation of epithelial mesenchymal transformation (EMT) in a variety of tumor cells. There are also highly active NR2F1 associated with neurodevelopmental diseases. The C5 subgroup also highly expressed the transcriptional factors TEAD4 and PKNOX1. TEAD4 is a key transcription factor in the Hippo signaling pathway, which participates in organ size control and tumor inhibition by limiting proliferation and promoting apoptosis (Shi et al., 2017, p. 4). The core of this pathway is composed of kinase cascades, in which MST1/MST2 recombines with its regulatory protein SAV1, phosphorylates, and activates LATS1/2 combined with its regulatory protein MOB1, and then phosphorylates and inactivates YAP1 oncoprotein and WWTR1/TAZ. It plays a role by mediating the gene expression of YAP1 and WWTR1/TAZ, thus regulating cell proliferation, migration, and EMT induction. Transcription factor PKNOX1 is involved in angiogenesis, suggesting that Schwann cells may promote the proliferation of VS by actively participating in tumor angiogenesis. These results suggest that Schwann cells play an important role in tumorigenesis and development (Figure 2F).

### Tumor immune microenvironment of sporadic vestibular schwannoma

**FIGURE 3:**
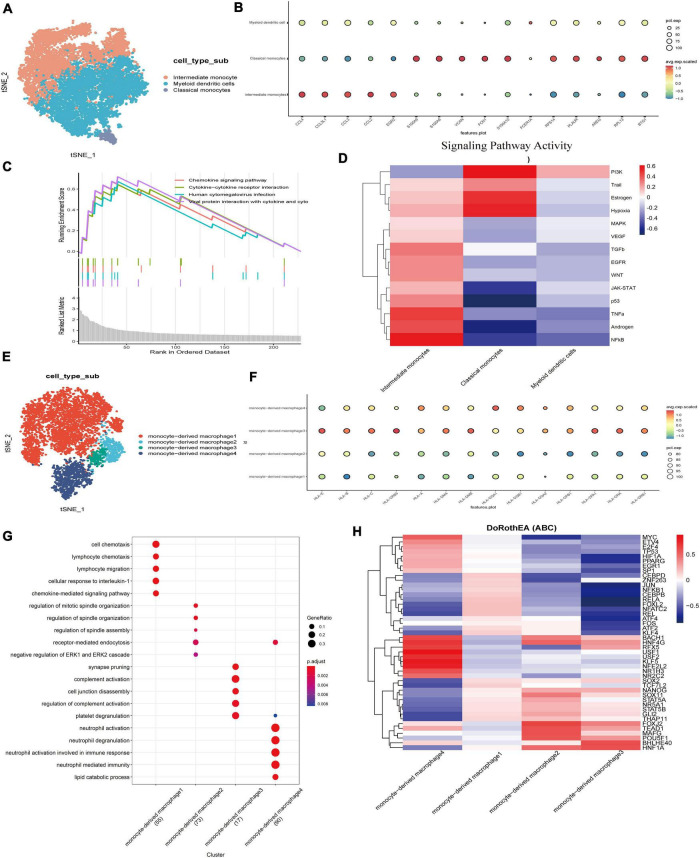
Transcriptome analysis of myeloid cells led to the detection of predominantly monocytes and macrophages with pathways involved in immune metabolism and inflammation.**(A)** TSNE showing myeloid cell subsets. Different cell types are indicated by different colors. **(B)** Three major cell types in myeloid cells were identified and annotated based on the expression pattern of canonical cell markers. **(C)** Pathway display of the top four in the GSVA enrichment score of myeloid cells. **(D)** Heatmap of top 50 highly variable TF activities among the myeloid cell subsets; the z-scores of TF activities are coded. **(E)** The UMAP of a new subgroup of intermediate monocytes. **(F)** Dot plots showing HLA gene scores for different macrophage populations. **(G)** Dot plots showing gene GO term enrichment in different macrophage populations. **(H)** Heatmap of top 50 highly variable TF activities among the Macrophage cell subsets; the z-scores of TF activities are coded.

Currently, emerging evidence shows that in addition to the proliferation of Schwann cells in the tumor microenvironment of VS, complex inflammatory responses also play a key role in its growth and proliferation (Lewis et al., 2019). Myeloid cells play an important role in resisting pathogen invasion and regulating inflammatory responses. The key functions are mediated by differentiated cells, including macrophages and dendritic cells. Monocytes can be divided into subgroups according to the presence of lipopolysaccharide (LPS) co-receptor CD14 and Fc-γ receptor III (CD16). We subdivided the myeloid cells into three subgroups. According to the classical cell marker genes, the clusters highly expressing CLEC9A, TXNIP, and AREG were defined as dendritic cells (Figures 3A,B; Collin and Bigley, 2018). GO enrichment revealed that the genes of the other two clusters were significantly different in functional characteristics (Supplementary Figure 2C). According to the literature, the clusters mainly involved in activating the nuclear factor kappa-B (NF-κB) pathway, rheumatoid arthritis, and sarcoidosis were defined as intermediate monocytes, while another group of classical monocytes highly expressed genes such as S100A8, S100A10, and VCAN (Supplementary Figure 2E; Nahrendorf and Swirski, 2016). The GSEA analysis of differential genes showed that the top four enrichment pathways were the interactions of cytokines with cytokine receptors and the chemokine signaling pathways, and the interactions of viral proteins with cytokines and cytokine receptors, suggesting that monocytes may act on inflammatory, immune, and viral infection-related pathways and are in an activated immune state (Figure 3C). Through the tumor pathway score, we found that the intermediate monocytes had higher scores of NF-κB, hypoxia, EGFR, and TGF-β signal transduction pathway activity, while classical monocytes (Figure 3D) had higher PI3K signal transduction scores (Supplementary Figure 2D). The PI3K/ATK pathway is a signal transduction pathway that is continuously activated in tumor cells, which can lead to excessive proliferation and inhibit apoptosis of tumor cells. Studies have found that this pathway plays a role in chemotherapy resistance by promoting proliferation and inhibiting tumor cell apoptosis in a variety of tumors (Liu et al., 2020).

It is not clear whether intermediate monocytes represent a truly different monocyte cluster, or whether it is just a transitional phase between classical and non-classical cells. The intensity of the NF-kB signaling pathway of intermediate monocytes was significantly higher than that of other clusters. Previous studies have shown that activated NF-κB is the mechanism responsible for the upregulated expression of p75NTR to promote the survival of Schwann cells (Zhang et al., 2021). High NF-κB activity enhances hepatocyte growth factor (HGF) and the c-Met autocrine feedforward loop to promote tumor cell proliferation and increases the expression of prognostic factors in VS, such as matrix metalloproteinase 2 (MMP-2), MMP-9, MMP-14, COX-2, interleukin 1 (IL-1), IL-6, TNF- α (Xu et al., 2019), and signal transduction transcriptional activator 1 (STAT1) (Solinas et al., 2009), or the absolute tumor growth rate of VS (Behling et al., 2019).

To further clarify the characteristics of intermediate monocytes, intermediate monocytes were extracted and reclustered, and 2,322 cells were subdivided into four groups (Figures 3E,G) by functional enrichment analysis. The first 30 genes were positively or negatively correlated with the principal component PCA1, and the highly expressed genes in the 500 cells with the highest or lowest PCA score were displayed (Supplementary Figure 2F). A group of cells was shown to highly express CCL4L2, CCL4, and other chemokines and their receptors as well as IL1B, indicating the function of promoting tumor growth. In contrast, another group was found to highly express GPNMB, HLA-DRB5, HLA-DRB6, and other genes that highly encode MHC class I and II molecules, which specifically present antigens to cytotoxic CD8 T lymphocytes. MHC class II is a glycoprotein present in specific antigen-presenting cells (including macrophages). We examined the expression of MHC class II by evaluating the level of the gene HLA family. The increased expression of MHC class II in monocyte-derived macrophage 3 and monocyte-derived macrophage 4 indicates that macrophages have changed to an activated state, in which they have acquired the ability to present tumor-specific antigens (Figure 3F). M2 macrophages mainly secrete cytokines such as IL-10 and TGF-β to inhibit inflammation as well as T cell proliferation and differentiation and promote tumor cell proliferation and tumor matrix angiogenesis. Other experiments have shown that macrophages in the nerve bridge initiate the formation of new blood vessels by releasing VEGF-A. In the process of nerve bridge formation, these new blood vessels provide structural support for the migration of Schwann cells and guide the growth of regenerated axons along them (Cattin et al., 2022).

To identify different key transcription factors in different states of macrophages and compare the differences in regulators, single-cell regulatory network inference and clustering were used to draw a transcription factor activity heat map (Figure 3H). Based on the calculated transcription factor activity score, we can determine the difference in transcription factor activity between cell types. There were significant differences in macrophage clusters (Supplementary Figure 2G). The transcription factor activities of monocyte-derived macrophage 3 and monocyte-derived macrophage 2 were similar, while those of other macrophage clusters were significantly different. Monocyte-derived macrophage 4 highly expresses transcription factors such as MYC, USF1, and USF2. Among them, transcription factor USF1 has previously been reported to promote the invasion and migration of glioma cells by activating lncRNAHAS2-AS1. These results suggest that the differential expression of transcription factors and the differential activation of key signaling pathways are the potential mechanisms for the heterogeneity of the tumor immune microenvironment, which are worthy of our further study.

The results of tSNE subgroup clustering and SingleR annotation showed that T/NK subgroups existed. According to the marker genes, T/nature killer (NK) was further divided into four subgroups, namely CD4 + T (ISG15, IFI6), CD8 + T (GZMK), NK (GNLY), and CD8 + Native (IL7R) (Supplementary Figure 1F). The degree of infiltration of immune cells in VS tumors was different, but among the three tumor samples, CD8 + T was the largest population in the immune cell lineage (Supplementary Figure 1C).

### Complex intercellular and molecular interaction networks in vestibular schwannoma

**FIGURE 4:**
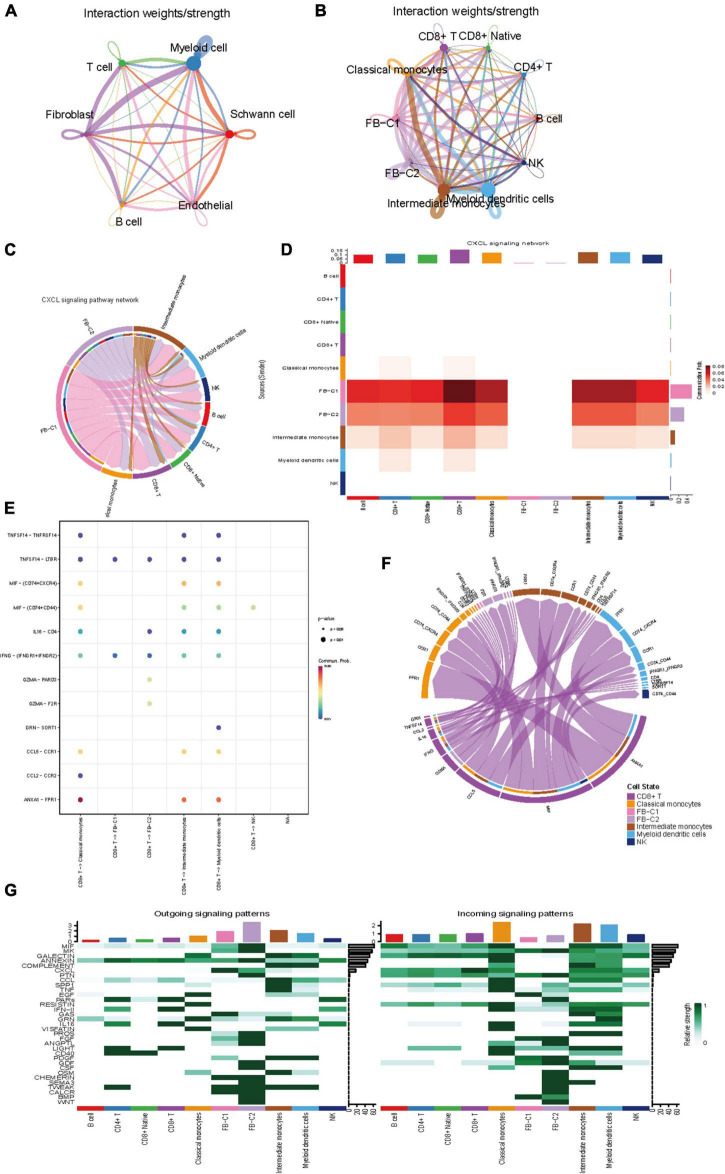
Intercellular communication between cell subset in VS. **(A)** Circle plot showing the intercellular communication between major VS cell types, using Cellchat workflow. The lines originating from a cell type indicate ligands being broadcast, with these lines connecting to the cell type where the receptors are expressed. The thickness of the line is proportional to the number of unique ligand-receptor interactions, with loops representing autocrine circuits. **(B)** The network between Subpopulations of T cells, B cells, myeloid cells and fibroblasts. **(C)** The CXCL signaling pathway network. **(D)** Heatmap of differential interactions between Subpopulations of T cells, B cells, myeloid cells and fibroblasts in the cell-cell communication network of the CXCL signal pathway. The top-colored bar plot indicates the sum of column values (incoming signaling), and the right bar plot indicates the sum of row values (outgoing signaling). Red indicates increased signaling in CXCL pathway, and blue indicates decreased signaling. **(E)** The CXCL signaling pathway ligand receptor bubble plot. **(F)** Communication string diagram of cell subsets. **(G)** Signaling role analysis on the cell-cell communication networks of interest.

To describe the molecular connections behind cell-to-cell relationships, CellPhoneDB analysis was used to identify molecular interactions between ligand-receptor pairs and major cell types to build cellular communication networks. First, CellPhoneDB was used to analyze the interaction between six types of cells (Figure 4A). There was a significant relationship between fibroblasts and immune cells. The key signal events (Figure 4B) between fibroblast clusters and immune cell clusters were further predicted, and the results revealed the interaction between fibroblast clusters and immune cell clusters of ligand receptors. In the CXCL signal pathway, FB-C1 and FB-C2 are the main signal senders, through which other clusters (Figure 4C) in the immune environment are regulated. Previous studies have shown that the chemokine family is closely related to tumor invasion, lymphatic metastasis, and distant metastasis. Tumor-associated fibroblasts can transform normal fibroblasts into malignant ones by secreting CXCL and promote angiogenesis in tumor tissues. In the regulation of fibroblasts to other cells, there are many interactions with CD8 + T and a strong regulatory intensity (Figures 4D,E). In the mutual regulation of other cells, CD8 + T and classical monocyte mainly rely on ANXA1-FPR1 receptor pairs to play a role (Figures 4F,G). ANXA1 is a Ca2 + -dependent phospholipid binding protein, which is involved in a variety of cellular pathophysiological processes. It has been found that ANXA1 is closely related to many types of malignant tumors, and it may be involved in the occurrence and development of VS tumors. In addition, it has been reported that ANXA1-FPR1 is generally upregulated in peripheral blood immune cells of critically ill patients during the onset of COVID-19, suggesting that critically ill patients may have a systemic immune response storm mediated by ANXA1-FPR1. ANXA1-FPR1 may play a role in the occurrence and development of vestibular schwannoma, and further research can be carried out based on this finding.

### Gene expression omnibus database verification

**FIGURE 5:**
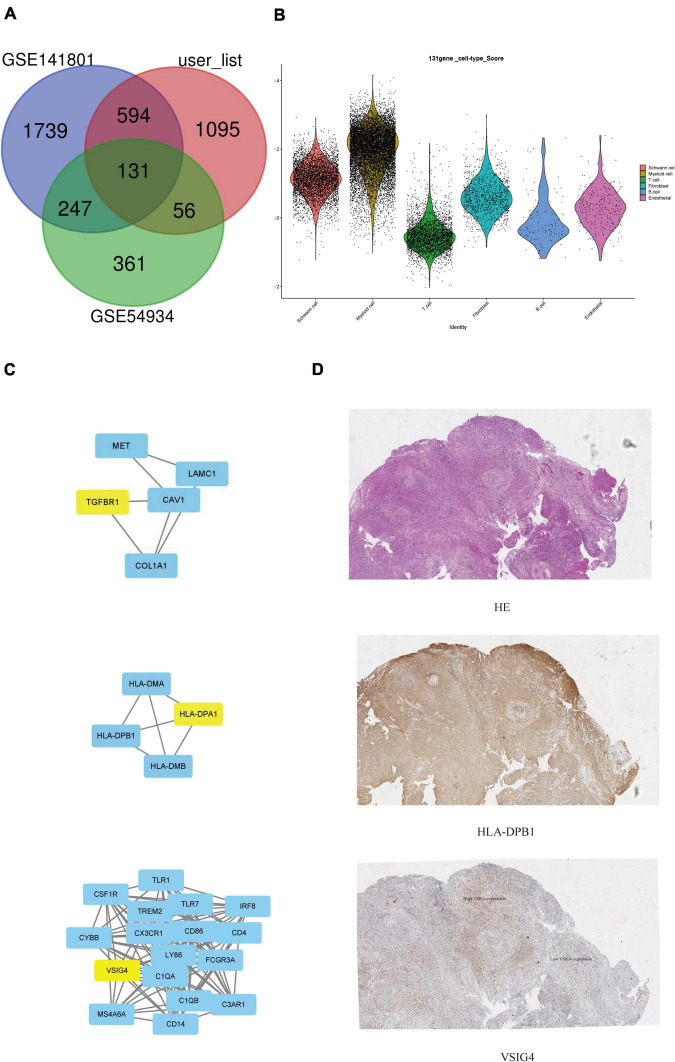
Search for potential biomarkers of VS in conjunction with gene expression omnibus (GEO) database. **(A)** Venn diagram of common specific genes. **(B)** Violin plots showed the scaled expression levels of the 131 overlapping genes in the sub-cluster. **(C)** Protein interaction network centered on hub gene. **(D)** Immunofluorescent staining of VSIG4 and HLA-DPB1 in paraffin-embedded VS tissue sections. The detected proteins using respective antibodies in the assays are indicated on the VS tumor.

We verified that sporadic VS-related datasets GSE141801 (36 sporadic vestibular schwannomas and seven normal tissue samples) and GSE54934 (31 sporadic vestibular schwannomas and three normal meningeal tissue samples) were downloaded from the GEO database. Sporadic VS and normal nerve tissue samples were selected. After standardizing the microarray results, DEGs (2,711 in GSE141801 and 795 in GSE54934) were identified. There are 131 overlapping genes between the three data sets, as shown in the Venn diagram (Figure 5A). The gene scores of the 131 differential genes in our data set were calculated and shown in the violin map, mainly in myeloid and Schwann cells (Figure 5B). To analyze the biological classification of the DEGs, DAVID was used for functional and pathway enrichment analysis. GO analysis showed that the changes in DEGs BP were significantly enriched in protein activation cascade, complement activation, defense response, mitotic cell cycle, and cell cycle process. The changes in molecular function (MF) were mainly concentrated in carbohydrate binding, oxidoreductase activity, mannose binding, scavenger receptor activity, and monosaccharide binding. The changes in cellular components (CC) of DEGs were mainly concentrated in the extracellular region, membrane attack complex, and chromosomes. KEGG pathway analysis showed that downregulated DEGs were mainly enriched in the cell phagocytosis, glycolysis/gluconeogenesis, and metabolic pathways, while upregulated DEGs were mainly enriched in the signal receptor pathway, MHC class II, and other pathways (Supplementary Tables 2, 3). A total of three genes were identified as hub genes with degree ≥ 10, namely HLA-DPB1, VSIG4, and TGFBR1 (Figure 5C). CBioPortal online platform was used to analyze the network of central genes and their co-expressed genes. These three hub genes suggest that they may play an important role in the occurrence or progression of VS. It has been found that in tumor microenvironment, VSIG4 is expressed on M2 macrophages or tumor-associated macrophages (TAM). After binding to ligands on CD8 + T cells, it inhibits the cytotoxicity of T cells, thus promoting tumor progression. After blocking the interaction between VISG4 and CD8 + T cells with VSIG4 antibody, it can relieve the functional inhibition of T cells, polarize M2 macrophages into M1 macrophages, stimulate the proliferation of CD8 + T cells, and thus inhibit tumor (Huang et al., 2019a, p. 4). Recently, it has also been reported that the expression of VSIG4 is related to the regulation of anti-tumor immunity, such as lung cancer and high-grade glioma (Liao et al., 2014, p. 4; Xu et al., 2015). Based on the results of previous analysis of immune infiltration of VS, we speculate that Immune-related genes VISG4 and HLA-DPB1 may be the targets of acoustic neuroma and verified by immunohistochemistry (Figure 5D).

## Discussion

VS is a histologically benign tumor, it is characterized by slow growth with a low probability of metastasis or malignant transformation, but its growth pattern can vary greatly depending on characteristics such as tumor size (Samii et al., 2017; Sethi et al., 2021). It can lead to brain stem compression and even death if the tumor continues to grow. At present, surgical treatment is the most recommended treatment for larger tumors, and targeted drug therapy was considered to be a new treatment direction to avoid trauma caused by surgery. Bevacizumab, everelimius and lapatinib are potential options for the treatment of VS. In addition, a link between tumor growth and non-steroidal anti-inflammatory drugs such as aspirin has been recognized. Bevacizumab is a mab and VEGF inhibitor (Shih and Lindley, 2006). However, long-term evidence of effectiveness is needed. In recent years, there has been some progress in the research on the mechanism of targets such as the NF2 gene and its encoded merlin protein, but it is still a long way from the precise control of VS. Recent studies have shown that some epidemiological, clinical, and radiological features are thought to be associated with a subsequent increased risk of tumor growth, such as the patient’s age, tumor location, first follow-up time, among others (Goshtasbi et al., 2020; Kim and Cho, 2021). Sporadic VS is not only the local proliferation of Schwann cells but should also be regarded as a complex immune microenvironment (Lewis et al., 2020). Data from isolated tissue specimens suggest that immune cell infiltration is an important component of the VS tumor microenvironment. However, the key molecular pathways driving this inflammatory microenvironment have not been identified (Hannan et al., 2020).

In this study, single-cell sequencing technique was applied to sporadic VS for the first time to characterize the cellular heterogeneity of sporadic VS at the single cell level. Using these single-cell data, we identified a total of six cell clusters. VS is mainly composed of myeloid cells and Schwann cells. The marker genes, specific gene characteristics, and microenvironment of Schwann cells and myeloid cells play a potential role in the occurrence and development of tumor, which provides a new research direction for the potential targeted drugs.

The expression profile and function of Schwann cells were heterogeneous, and each subgroup had a different degree of differentiation, proliferation, and immunocyte chemotaxis. Differentiation trajectories can reveal branching and linear differentiation processes, which are reconstructed by time sequencing of single cells (Trapnell et al., 2014). Herein, in our differentiation trajectory, SC-C5_myelin and SC-C6 cell clusters were located at the beginning of differentiation and differentiated into other Schwann cell clusters. It is worth noting that through the analysis of Schwann cell transcription factors, we found that Schwann cells may promote the proliferation of vestibular schwannoma by promoting vascular proliferation through transcription factor PKNOX1. It has been reported that the expression of VEGF, VEGFR-1/Flt, VEGFR-2/Flk, and coreceptor NP1 in VS tissue is significantly higher than that in normal vestibular nerve, suggesting that at least part of tumor growth is achieved by promoting angiogenesis (Zhang et al., 2021). This is consistent with our research results.

Monocytes and dendritic cells were mainly detected in myeloid cells, in which intermediate monocytes could further differentiate into M1 and M2 phenotypic macrophages. M2 phenotypic macrophages highly encode MHC class I and II molecules, suggesting that macrophages are in a state of activation. In addition, upregulated genes of myeloid cells have been found to play a prominent role in chemotaxis, especially the chemokine signaling pathway, cytokine-cytokine receptor interaction, and human cytomegalovirus infection. Cytokines often affect tumor cells directly or indirectly through inflammatory reactions, free radicals, and signal pathways, and play different roles in different immune stages, antigen treatment and the presentation of tumors. Cytokines are often the products of myeloid cells (Chung and Lim, 2014); therefore, myeloid cells have the potential to become drug therapeutic targets for sporadic VS.

Subsequently, we studied the interaction of ligand receptors between fibroblasts and immune cells. It was found that fibroblast clusters regulate other clusters of cells in the immune environment through the CXCL pathway. In addition, in the immune environment, CD8 + T and classical monocyte mainly rely on ANXA1-FPR1 receptor pairs to play a role (Vecchi et al., 2018, p. 1).

To further study the potential targets of drug therapy for vestibular schwannoma, we downloaded sporadic VS-related datasets GSE141801 and GSE54934 from the GEO database. The differential genes were identified, and 131 targeted genes were then obtained from the intersection of our own dataset, of which three genes were identified as hub genes and further verified in pathological sections by immunohistochemistry.

In conclusion, our work is the first study to explore the intratumor heterogeneity of sporadic VS tissues by scRNA-seq. Cells in the VS tissues were finely clustered into various groups, including Schwann cells. At present, many achievements have been made in the study of tumor growth and biomarkers. The occurrence and development of VS are widely related to genetic and epigenetic abnormalities of merlin, inflammatory factors, proteolytic enzymes, and nutritional supply pathways. Together with previous studies, our work might help in promoting the identification of the biomarkers of VSs and the exploration of the comprehensive tumor heterogeneity. Briefly, we delineated the landscape of sporadic VS and the intra-tumor heterogeneity, which could help researchers have a better understanding of the tumor progression.

## Data availability statement

The data presented in this study are included in the article/Supplementary material. Further inquiries can be directed to the corresponding author. The patients’ data are not publicly available due to ethical issues. Requests to access the datasets should be directed to CY, YIDIANCHU@gmail.com.

## Ethics statement

The studies involving human participants were reviewed and approved by the study of the project was approved by Lihuili Hospital of Ningbo University ethics committee (KY2020PJ191). The patients/participants provided their written informed consent to participate in this study.

## Author contributions

CY: conceptualization, methodology, software, investigation, formal analysis, and writing—original draft. LC: data curation and writing—original draft. DH: visualization and investigation. LY: resources and supervision. SZ: conceptualization, funding acquisition, resources, supervision, writing—review and editing. All authors contributed to the article and approved the submitted version.

## Footnotes

1
https://github.com/plger/scDblFinder

2
https://www.gsea-msigdb.org/gsea/msigdb/index.jsp

3
http://www.cbioportal.org

4
http://www.rproject.org

## Supplementary material

Supplementary Figure 1 **(A)** Parametric graph of quality control. **(B)** Absolute number of cells. **(C)** Violin plots showed the marker gene of the 6 subgroups. **(D)** GSVA analysis of gene. **(E)** Four major cell types in T cells were identified and annotated based onthe expression pattern of canonical cell markers. **(F)** Proportion of T cell subsets in different samples.

Supplementary Figure 2 **(A)** Dot plots showing gene GO term enrichment in different Schwann cell subpopulations. **(B)** The cell cycle of Schwann cell subsets. **(C)** Dot plots showing gene GO term enrichment in different monocyte subpopulations. **(D)** Volcano plot of differentially up/down-regulated genes. **(E)** Heatmap of the top 30 genes positively or negatively correlated with principal component 1 which were defined as the “M1 macrophages” and “M2 macrophages” gene signature, respectively, shown for the top 500 cells with the highest or lower PCA scores, respectively. **(F)** Dot plots showing gene GO term enrichment in different monocyte subpopulations.

Supplementary Figure 3 **(A)** Information on three patients. **(B)** Preoperative images of three patients. **(C)** Expression level of the three target genes in the sample.
