# Supplementary figures and images for: Single-cell sequencing reveals the cell map and transcriptional network of sporadic vestibular schwannoma
Source: Front Mol Neurosci. 2022 Oct 11;15:984529. doi: 10.3389/fnmol.2022.984529 (PMC9592810; doi:10.3389/fnmol.2022.984529)

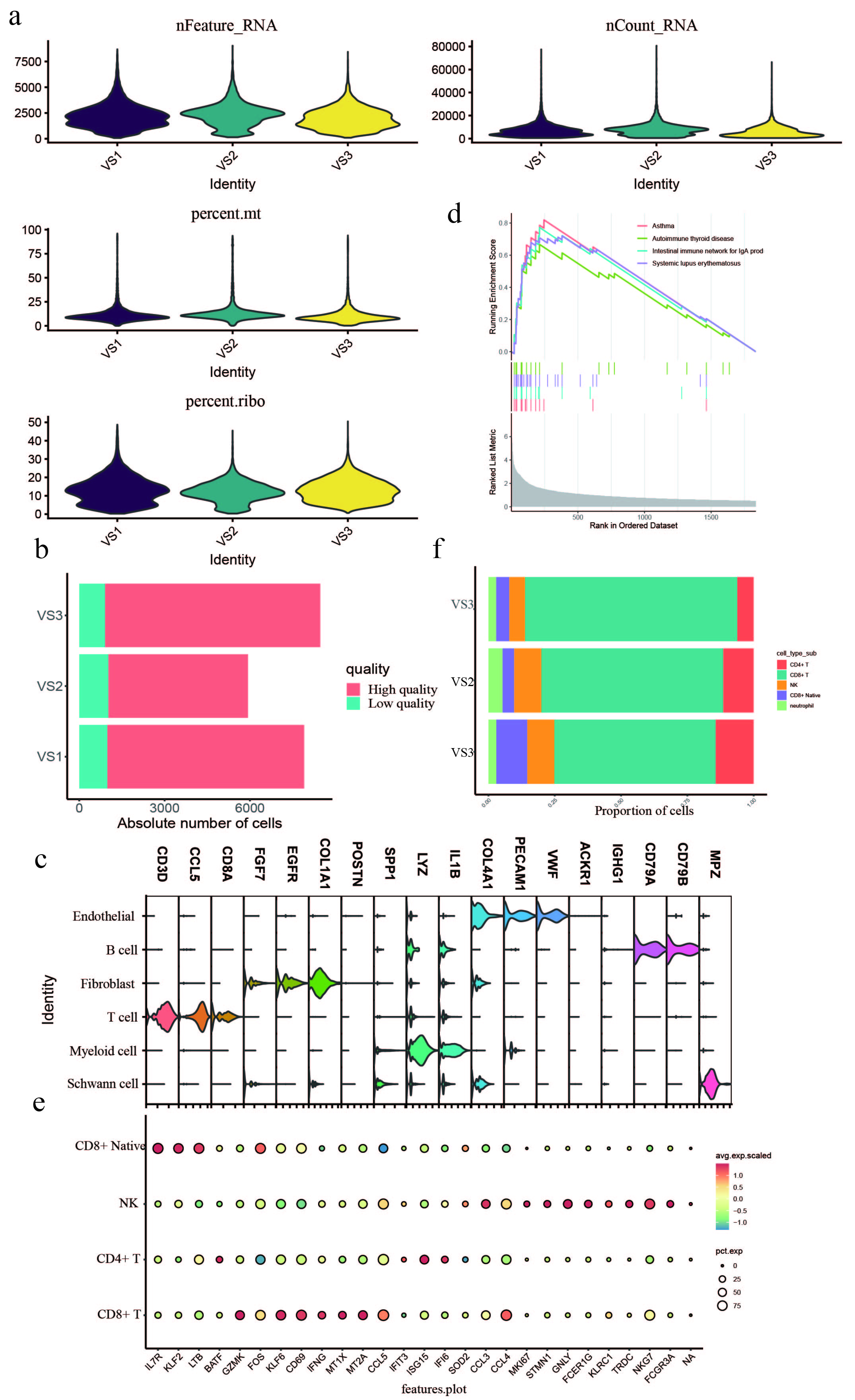

Supplement: Supplementary Figure 1 — (A) Parametric graph of quality control. (B) Absolute number of cells. (C) Violin plots showed the marker gene of the 6 subgroups. (D) GSVA analysis of gene. (E) Four major cell types in T cells were identified and annotated based onthe expression pattern of canonical cell markers. (F) Proportion of T cell subsets in different samples. [file Image_1.jpg]

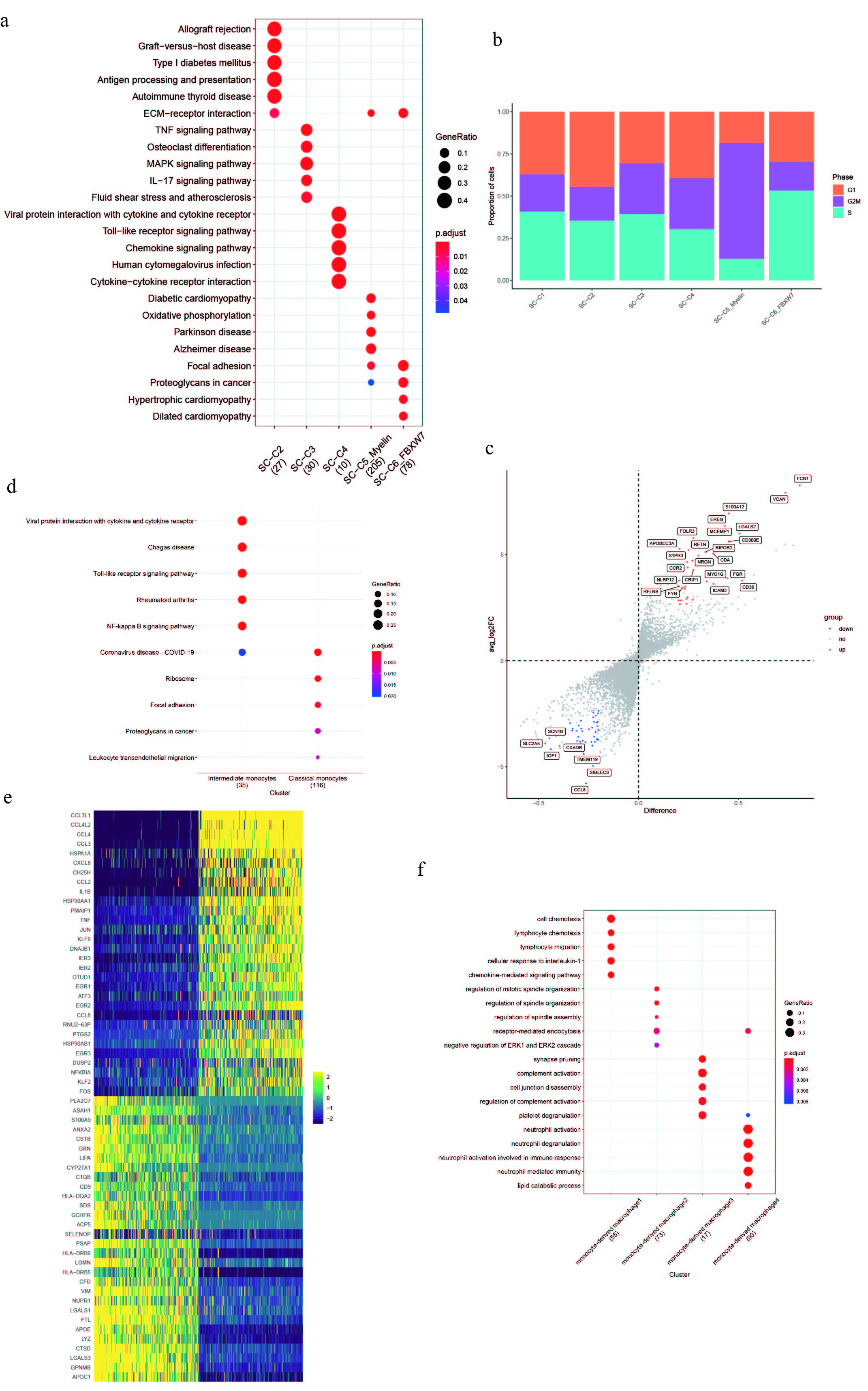

Supplement: Supplementary Figure 2 — (A) Dot plots showing gene GO term enrichment in different Schwann cell subpopulations. (B) The cell cycle of Schwann cell subsets. (C) Dot plots showing gene GO term enrichment in different monocyte subpopulations. (D) Volcano plot of differentially up/down-regulated genes. (E) Heatmap of the top 30 genes positively or negatively correlated with principal component 1 which were defined as the “M1 macrophages” and “M2 macrophages” gene signature, respectively, shown for the top 500 cells with the highest or lower PCA scores, respectively. (F) Dot plots showing gene GO term enrichment in different monocyte subpopulations. [file Image_2.jpg]

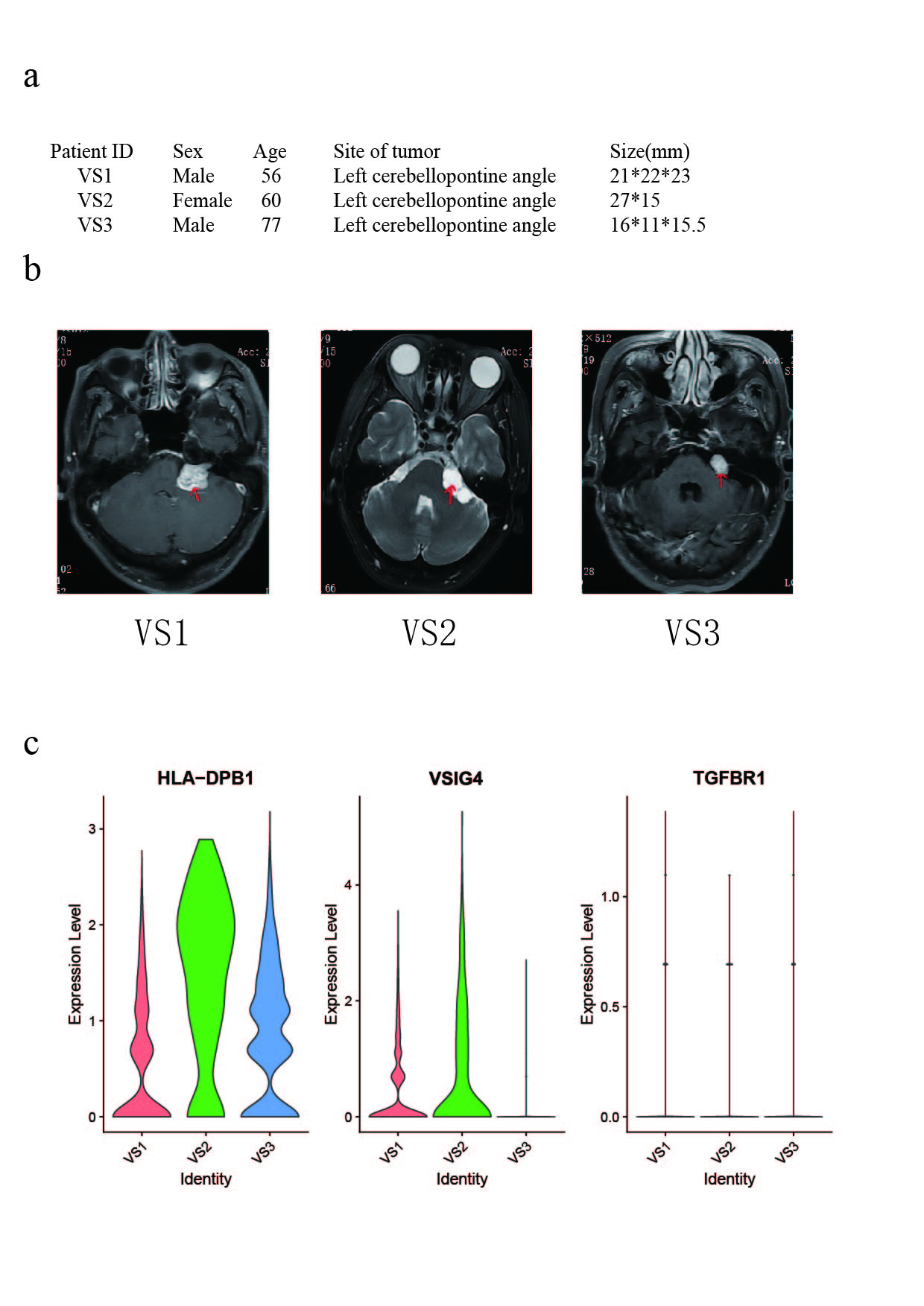

Supplement: Supplementary Figure 3 — (A) Information on three patients. (B) Preoperative images of three patients. (C) Expression level of the three target genes in the sample. [file Image_3.jpeg]
